# The Lysine 65 Residue in HIV-1 Reverse Transcriptase Function and in Nucleoside Analog Drug Resistance

**DOI:** 10.3390/v6104080

**Published:** 2014-10-23

**Authors:** Scott J. Garforth, Chisanga Lwatula, Vinayaka R. Prasad

**Affiliations:** Department of Microbiology and Immunology, Albert Einstein College of Medicine, Bronx, NY 10461, USA

**Keywords:** HIV-1 reverse transcriptase, NRTI analog resistance, K65R mutation, HIV-1 drug resistance, reverse transcriptase function, polymerase fidelity

## Abstract

Mutations in HIV-1 reverse transcriptase (RT) that confer nucleoside analog RT inhibitor resistance have highlighted the functional importance of several active site residues (M184, Q151 and K65) in RT catalytic function. Of these, K65 residue is notable due to its pivotal position in the dNTP-binding pocket, its involvement in nucleoside analog resistance and polymerase fidelity. This review focuses on K65 residue and summarizes a substantial body of biochemical and structural studies of its role in RT function and the functional consequences of the K65R mutation.

## 1. Introduction

Human immunodeficiency virus type 1 (HIV-1) reverse transcriptase (RT) continues to be a major target of HIV therapies. Biochemical and structural studies of mutations that confer resistance to RT inhibitors (RTIs), specifically to the nucleos(t)ide analog RT inhibitors (NRTIs), have provided a considerable degree of mechanistic insight into the role of key amino acids in the dNTP-binding pocket and their role in NRTI-resistance, catalysis, dNTP selection and polymerase fidelity. Resistance to NRTIs is conferred via two distinct mechanisms. Mutations conferring resistance to thymidine analog class of NRTIs such as 3'-azido-3'-deoxythymidine (AZT), 3’-deoxy, 2’, 3’-didehydrothymidine (d4T) do so by facilitating excision of their monophosphate versions (AZTMP and d4TMP respectively) from the growing chain of DNA in a reaction that is akin to the pyrophosphorolysis reaction [1,2]. It has been proposed that such mutations create a surface for binding ATP, which serves as the phosphate donor to regenerate AZTTP in the excision reaction [3]. A second class of resistance mutations confer the ability to discriminate against the NRTI analog, while not affecting the incorporation of the normal dNTP substrate (exclusion mechanism). This class of mutations is exemplified by M184V, Q151M and K65R mutations. The M184 residue, which forms a part of the highly conserved signature motif in all RTs — YXDD (YMDD in HIV-1 RT), is mutated to a valine in response to Lamivudine (2’-deoxy-3’-thiacytidine; 3TC) or Emtricitabine (2’,3’-dideoxy-5-fluoro-3’-thiacytidine; FTC) treatment [4]. In the 3-dimensional structure of RT bound to template-primer and dNTP, this residue is located in the polymerase primer grip near the active site adjoining two of the aspartates that are part of the catalytic aspartate dyad [5]. Biochemical and structural studies of this variant of RT have yielded important insights into how M184V alteration confers discrimination between the normal dNTP substrate and the NRTI analog, 3TC triphosphate (3TCTP) through the exclusion mechanism [6]. Q151 forms part of the LPQG motif that is well-conserved in retroviral RTs. Q151 is mutated to methionine (Q151M) in response to treatment with dideoxynucleoside analogs and usually occurs in combination with A62V, V75I, F77L, and F116Y mutations [7]. The Q151M complex of mutations confers resistance to multiple NRTIs by allowing the enzyme to discriminate against the NRTI analogs [8,9,10]. Together with residues D113, A114, Y115 and F116, Q151 forms a 3’ ‘pocket’ that interacts with the 3’-OH of the dNTP substrate [11]. The K65 residue, the main focus of this review, is discussed below.

## 2. K65 in HIV-1 RT Function

K65 of HIV-1 RT has a major role in both drug resistance and polymerization fidelity. This residue is the site of important drug resistance mutations, resulting in decreased sensitivity to many of the nucleoside RT inhibitors (NRTIs). The strategic location of this residue in the nucleotide-binding pocket and its role in both fidelity and NRTI-resistance has stimulated a number of biochemical, structural and virological studies.

HIV-1 RT is a heterodimer, composed of subunits p66 and p51. In this chapter, we will be discussing the K65 residue present in the larger subunit, p66, which contains the polymerase active site. K65 is part of a run of basic residues in the flexible loop between the β3 and β4 strands of the dynamic “finger” region, critical for the polymerization reaction. Upon binding of a nucleotide, the fingers close over the catalytic site, forming a “closed complex” that is competent for catalysis (nucleophilic attack, formation of the phosphodiester bond and release of the pyrophosphate leaving group), following which the fingers subdomain moves away from the active site, returning to the “open complex” [11]. The nucleotide-binding pocket of HIV-1 RT consists of both protein and nucleic acid surfaces. K65 forms a part of the nucleotide binding pocket, and contributes to binding and correct orientation of the incoming dNTP; specifically, epsilon-amino group of K65 forms a salt bridge with the γ phosphate of the incoming nucleotide [11]. This stabilization is largely independent of the particular nucleotide to be incorporated; the steric fit of the base within the active site and hydrogen bond formation determine polymerase fidelity. Consistent with this role, it has been shown that mutant RTs in which the K65 is replaced with a non-basic residue, or deleted, exhibit higher fidelity as the role of the template sequence-determined interactions become proportionally more important [12,13]. This stabilization effect extends to binding, incorporation and therefore inhibition by NRTIs; as these analogs differ in their shape from the natural nucleotide substrate, the binding interactions through the invariant γ phosphate become of increased significance. The NRTIs are delivered to the cell as 2’-3’ dideoxynucleotide nucleosides (with the exception of tenofovir, which is a nucleoside phosphonate); are phosphorylated by cellular kinases and compete with the natural cognate nucleotide to block polymerization by acting as chain terminators. The role of substitutions of K65 in NRTI resistance is discussed in more detail below.

## 3. K65 in Nucleoside Analog-Resistance

The K65R mutation, which perturbs the geometry of the nucleotide bound in the active site, while retaining the stabilization of the cognate nucleotide required for polymerization, was identified in samples from patients who had undergone treatment with the 2’-3’dideoxynucleoside inhibitor ddC [14,15]. Subsequent *in vitro* cellular assays demonstrated that while this mutation lead to cross-resistance to the dideoxynucleoside inhibitors didanosine (ddI), ddC and Lamivudine (3TC), there was no effect on AZT sensitivity [14]. It is now known that K65R is associated with resistance to most of the NRTI drugs, including Abacavir (ABC), Emtricitabine (FTC), Lamivudine (3TC), Stavudine (d4T) and, in particular, Tenofovir [16,17,18,19]. *In vitro* experiments with purified recombinant RT carrying the K65R mutation recapitulated cross-resistance to the dideoxynucleoside inhibitors, and showed only a modest effect on AZT resistance [20].

The K65R mutant enzyme primarily confers resistance through the exclusion (discrimination) mechanism. Pre-steady state kinetic studies have shown an overall reduced efficiency of NRTI incorporation compared to the cognate nucleoside triphosphate. The decreased rate of NRTI incorporation (K_pol_) with a slight effect on the natural substrate leads to an increase in discrimination against the NRTI [20,21,22].

Cell-based *in vitro* studies demonstrating an absence of cross-resistance between AZT-resistance mutations and mutations leading to resistance to other, non-azido dideoxynucleotides [14,23] suggested that the mechanism used by HIV-1 for AZT-resistance could be distinct from the mechanism for resistance to other 2’-3’ dideoxynucleoside drugs. In groundbreaking work, it was demonstrated that the mechanism for AZT resistance was actually through excision of the incorporated AZTMP, rather than selection against incorporation at the level of binding to nucleotide-binding site, and that clinical AZT resistance is due to mutations that increase the efficiency with which AZTMP is excised after its incorporation by RT. The AZTMP is excised through an ATP-dependent phosphorolysis [1], resulting in the removal of the AZT monophosphate from the nascent DNA chain, and release of the dinucleoside tetraphosphate Ap4AZT. Several mutations (the thymidine analog mutations, TAM) are required for high-level AZT resistance by excision, and include M41L, D67N, K70R, T215F or Y and K219E or Q [24,25]. In addition to their effect on AZT, the TAMs have also been shown to increase excision of tenofovir; in both AZT and TFV resistance, the TAMs have little to no effects on the rate of binding or incorporation of the analog [26].

In the presence of physiologically relevant concentrations of ATP [27], pyrophosphate (PPi) [2], or inorganic phosphate [28], HIV-1 RT can unblock NRTI-monophosphate (NRTI-MP) terminated primers. The K65R has been shown *in vitro* to decrease the rate of pyrophosphorolysis [22] and is predicted to reduce ATP-mediated excision of AZTMP. The decreased rate of excision is attributed to the conformational constraints of K65R and Arg72 [29], as described in the following section. The mechanism of nucleoside analog resistance mediated through K65R mutation, however, is exclusively through selection against incorporation, rather than an increase in excision. Indeed, as described below, the K65R mutation is antagonistic to resistance occurring through post-incorporation excision [26,30].

## 4. Antagonism of K65R with other NRTI-Resistance Mutations

Although K65R confers cross-resistance only to NRTIs other than AZT, it actually increases the susceptibility of virus containing the TAM mutations *i.e.*, K65R is antagonistic to resistance via TAMs [26].

**Figure 1:**
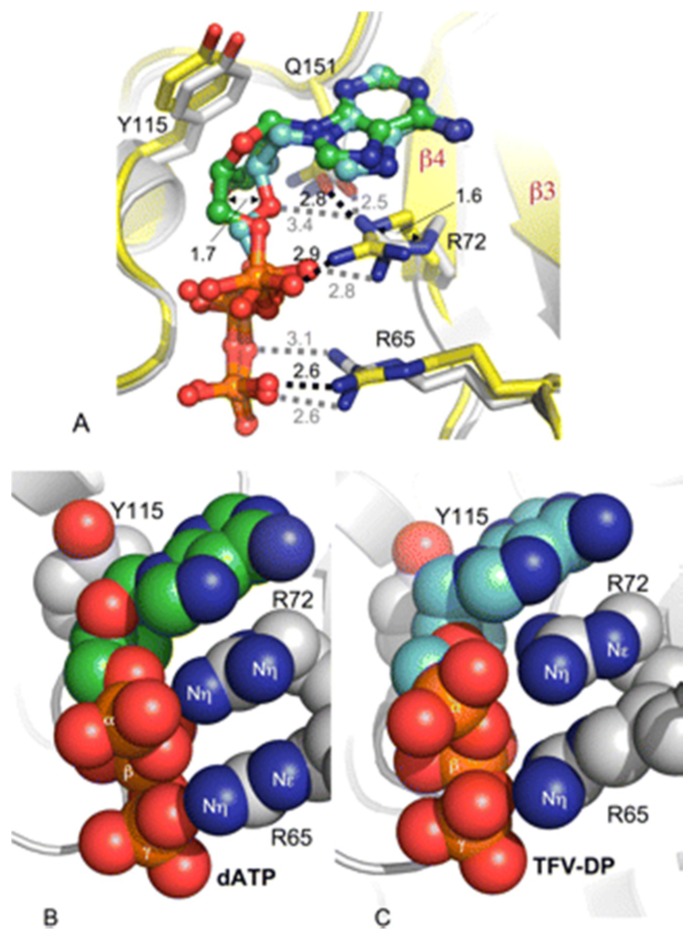
Comparison of binding of tenofovir diphosphate (TFV-DP) and dATP to K65R RT·dsDNA. *A*, overlay of TFV-DP (TFV-DP (*cyan*) and RT (*gray*) and dATP (dATP (*green*) with RT (*yellow*)) shows that the structural difference between the inhibitor and the substrate at the deoxyribose moiety positions Arg72 and Arg65 side chains differently. The polar interactions are represented by *dotted lines*. Shown is stacking of the guanidinium plane of Arg65 and guanidinium plane of Arg72 and adenine base in K65R RT·dsDNA·dATP (*B*) and K65R RT·dsDNA·TFV-DP (*C*) ternary structures. Despite differences in the rotameric conformations of Arg65 and Arg72, the hydrophobic stacking is maintained in both structures. *Reproduced from* [29].

The structural basis for both the broad dideoxynucleotide resistance and the AZT resistance antagonism by K65R has been elucidated [29]. The K65R substitution, although conservative in nature, has the effect of reducing the steric flexibility of the RT active site by the formation of a molecular platform through the stacking of the guanidinium planes of the R65 and R72 residues. R72 is an active site residue that in the wild-type enzyme is engaged in multiple interactions with the incoming nucleotide; it hydrogen bonds with the α and β phosphates, and stacks with the nucleotide base. In the K65R mutant RT, both the R72 and R65 residues are restrained due to the guanidinium stacking of the arginines, and an additional stacking interaction between the R72 guanidinium and the incoming base (see Figure 1). The structural data suggests that the reduced polymerization rate of the K65R mutant could be due to this steric constraint. When tenofovir (TFV-DP) is bound in the active site, the R72 is locked into an alternate rotamer, distinct from the conformation seen with a dNTP. The alternate rotamer allows a distinct pattern of guanidinium stacking, and may restrict flexibility further. Ultimately, the stacking interaction between the R65, R72 and base of the incoming nucleotide is hypothesized to impose such a steric constraint that NRTIs cannot achieve the conformation necessary for incorporation in virus carrying the K65R mutation [29]. This mechanism for the reduction in the rate of polymerization (k_pol_) is distinct from that of other NRTI resistance mutations, such as M184V that confer resistance by decreasing the binding affinity of the enzyme for the inhibitor [31].

Although the steric restraint imposed by the stacked guanidinium planes suggests a mechanism for resistance to nucleoside analogs, it does not explain the antagonistic relationship between K65R and the TAM mutations. The structure of HIV-1 RT in complex with the product of AZT excision (Ap4AZT) reaction was determined by Tu *et al*. [3], which revealed that although the TAM substitutions did not affect the binding interactions with the AZT moiety, compared to the wild-type, there was a substantial difference in interactions involving ATP, which catalyzes the AZT excision reaction. Two of the canonical TAM mutations, K70R and T215Y, form a new ATP binding pocket, which is not present in the wild-type enzyme. The ATP binding is further stabilized by the presence of secondary TAM mutations. The Arg70 mutant residue is responsible for binding and positioning the ATP, where the guanidinium group of Arg70 forms hydrogen bonds with the ATP α phosphate and the ribose 5’ O and 3’OH [3]. However, analysis of the structure demonstrates that the K65R-R72 guanidinium stacking, the basis of the mechanism for K65R dependent resistance to NRTI, would interfere with the positioning of ATP by Arg70 [3]. Thus, the K65R mutation appears to reduce the efficiency with which AZT is removed from the primer terminus.

Therefore, it is not surprising that the K65R mutation occurs very rarely in conjunction with the TAM mutations given that there is no enhanced NRTI resistance as a result of K65R [32]. Additionally, and a further reason for the rarity of TAMs and K65R together in a clinical setting, the antagonistic effect of K65R and TAM was shown to be bidirectional; the presence of the TAM mutations increases sensitivity to tenofovir in viruses containing the K65R substitution [30]. This bidirectional antagonism has been demonstrated most recently in a study using single genome sequencing, whereby plasma samples with K65R mutation in the presence of TAMs were sequenced. Data from this study shows that while K65R was present in 82% of genomes without TAMs, and at low frequency in the presence of <3 TAMs; no sequences were identified with K65R, T215F/Y and ≥ 2 TAMs in the absence of the Q151M multi-drug resistant complex [32]. This lack of linkage between TAMs and K65R *in vivo* aligns with the functional antagonism of the mutants for each other as shown by biochemical studies. The Q151M complex consists of five mutations (Q151M/A62V/V75I/F77L/F116Y), of which Q151M is one of the primary mutations to emerge. The addition of the other four mutations to the complex significantly increases resistance to AZT, ddI, ddC through a decrease in the rate of polymerization (k_pol_). The association of Q151M and other Q151M complex mutations with K65R, T215F/Y and other TAMs suggests that the Q151M complex is necessary to confer NRTI resistance in the presence of K65R and TAM antagonism [32].

## 5. K65R Confers Hypersusceptibility to other NRTIs

A relatively newer class of NRTIs includes translocation-defective RTIs (TDRTIs). The adenosine analog 4’-ethynyl-2-fluoro-2’-deoxyadenosine (EFdA), belongs to this category and inhibits RT by acting as a chain terminator or delayed chain terminator [33]. HIV-1 can be inhibited 10-times more efficiently by EFdA than TDF. Michailidis *et al*. have shown that K65R mutant virus can be inhibited 70-times more efficiently by EFdA than by TDF [34]. Investigations into the mechanistic basis of this hypersusceptibility showed the K65R mutation did not significantly enhance the susceptibility to EFdA either by affecting its incorporation or by affecting enzyme translocation on EFdA-MP-terminated template/primers. Interestingly, the K65R mutation appeared instead to cause hypersusceptibility by decreasing the rate of excision of EFdA from the primer termini. These authors have proposed that the structural basis for decreased excision may be due to the fact that R65 residue interfaces with R72 and the phosphate moieties of EFdA-MP thus affecting the nucleophilic attack of the phosphate donor (either pyrophosphate or ATP) on the EFdA-terminated primers [34].

## 6. Clinical Prevalence of K65R

In Europe and the USA, the K65R mutation is rarely observed clinically, although its frequency has increased over the last decade in parallel with the increased usage of tenofovir, as K65R is clinically associated with tenofovir resistance [35,36,37,38]. Even with tenofovir treatment, the occurrence of K65R in the patient population is typically below 5% [39]; however, as described below, this frequency is increased depending on HIV-1 subtype, geographical location and treatment regimen. The lack of translation of significant *in vitro* resistance to clinical observation may be due to the effect of the substitution on polymerization using natural substrate nucleotides, with a resulting decrease in viral fitness [40,41]. Accumulation of the K65R mutation has been shown to decrease the overall catalytic efficiency of HIV-1 RT up to 3-fold, almost entirely due to a reduced rate of incorporation (k_pol_) [40,42]. In addition, the decrease in tenofovir susceptibility of the K65R mutant has been associated with a reduction in RT processivity *in vitro* [40]. Studies using purified and virion associated RTs on stretches of homopolymeric and heteropolymeric templates show decreased processivity for K65R RT under limiting substrate concentrations [40,43]. The combined effects of the mutation on incorporation rate and processivity would clearly be expected to reduce viral fitness.

Further, the K65R substitution has an effect on polymerase fidelity; incorporation of an incorrect nucleotide into a nascent DNA chain requires both misinsertion and the extension of the resulting mismatched primer. Work from our group demonstrated that the efficiency of both these steps of misincorporation are reduced by the K65R mutation; the initial misinsertion showing a decreased catalytic rate, and primer misextension, through decreased nucleotide binding [12]. Furthermore, studies to measure the impact of this substitution on the overall mutation rate of HIV-1 RT using lacZα as a reporter gene *in vitro* revealed an 8-fold reduction compared to wild type. When the nucleotide sequence of lacZα mutants thus generated by K65R was analyzed, a uniform reduction in most types of RT errors, including substitutions and frameshifts, was noted [44]. This effect on fidelity may result in a reduced viral fitness as the viral population becomes less able to adapt to immune surveillance and drug therapy. There is evidence that other residues in this region of the fingers subdomain (ß3–ß4) also play an important role in fidelity. For example, biochemical studies of substitutions at the adjoining K66 have indicated that K66, much like K65, is a determinant of both dNTP misinsertion and mismatch extension efficiency [45].

Other substitutions have been described (K65E and K65N), but are much less common than K65R [35,36]. These substitutions also play a role in NRTI resistance through a decrease in binding affinity (K_m_) for dNTP substrates, reduced phosphorolysis and catalytic efficiency [22,46], however, are presumably less frequent as they have an even more dramatic effect on viral fitness than K65R.

Given the importance of the K65 residue in HIV-1 RT function and the observed reduced fitness of K65R mutants, it is no surprise that compensatory mutations would evolve to counteract this defect. Two mutations in particular, A62V and S68G, have been found in association with K65R sequence databases and *in vivo*. While the double (A62V/K65R, S68G/K65R) or triple mutants (A62V/K65R/S68G) do not grossly affect NRTI (TFV, FTC, 3TC, ddI or d4T) susceptibility, they appear to improve the viral fitness by restoring the nucleotide incorporation efficiency (k_pol_/K_d_) to wild type levels, and partially restoring NRTI excision [47]. In one study, virus from 50% of patients (4/8) who developed the K65R mutation following tenofovir treatment also contained at least one of the A62V or S68G mutations; however, in all patients with the K65R virus, viremia did not return to the pre-treatment baseline, potentially reflecting the effect of K65R on viral fitness [48].

## 7. Subtype Differences

HIV-1 is classified into a number of groups—M (main group), N (the non-M), O (outlier) and P. The M group of HIV-1 isolates cause most of the HIV-1 infections worldwide and is further divided into 9 subtypes (A, B, C, D, F, G, H, J and K). Subtype B is the virus responsible for the majority of infections in the western hemisphere; however, globally, subtype C is responsible for more than 50% HIV-1 infections [49]. The average sequence diversity for the pol open reading frame, which encodes the RT, across the subtypes is 10%, allowing for the possibility that there could be variability in function, drug sensitivity, and selection of resistance mutations [50].

**Figure 2:**
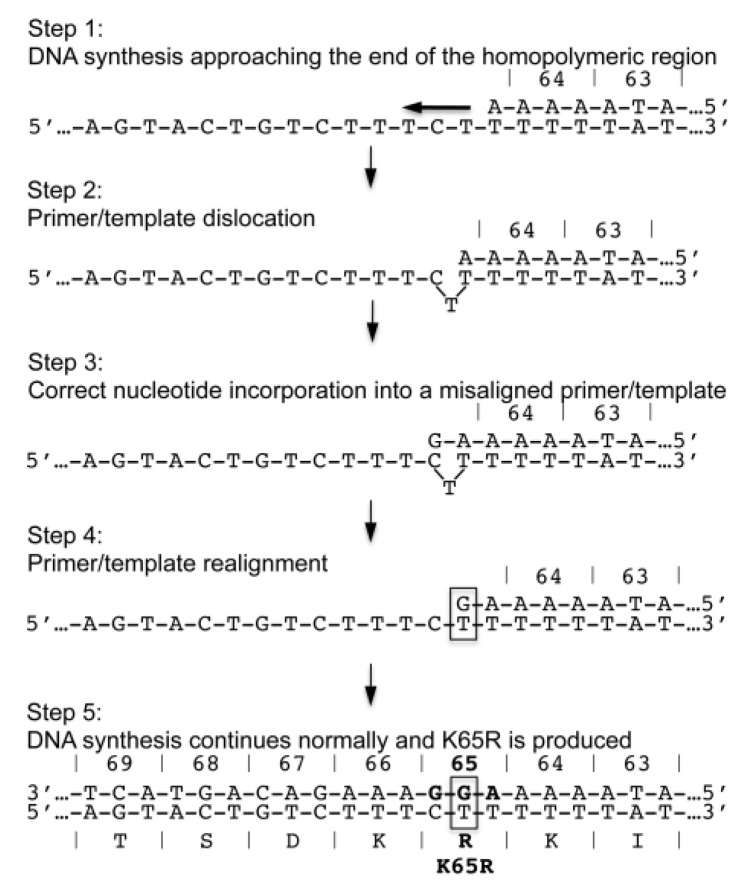
Schematic of dislocation mutagenesis and the development of the K65R mutation in subtype C HIV-1. *Step 1*: DNA synthesis approaches the end of a homopolymeric nt stretch that ends precisely at the location of K65R development. *Step 2*: At the end of the sequence, the RT enzyme exhibits characteristic pausing of DNA synthesis. The template-strand folds onto itself and exposes a C in the folded-over template strand. *Step 3*: A dGTP nt correctly binds opposite the C base of the misaligned template strand as DNA synthesis continues. *Step 4*: The primer and template strands realign and the same C base becomes re-exposed on the now correctly aligned template strand. *Step 5*: A second dGTP becomes incorporated opposite the re-exposed C on the correctly aligned primer/template strands and DNA synthesis continues normally. This series of events yields the AAG-to-AGG change that is responsible for the more facilitated appearance of the K65R mutation in subtype C HIV-1. *Reproduced from* [55]*.*

In a finding that could indicate that subtype differences can inform treatment choices, it was discovered that the K65R mutation is selected rapidly in subtype C viruses in cell culture as a result of tenofovir treatment. While K65R emerged after 12 weeks in subtype C, it was not detected in subtype B virus treated with the same regimen for up to 75 weeks [51]. The simplest explanation for the finding was that differences between the amino acid sequence of the RTs from the two subtypes of HIV-1 may be responsible for the difference in mutation selection (such as presence of compensatory mutations in subtype C to overcome the inherent reduction in RT activity due to the K65R substitution). This was demonstrated not to be the cause of this difference [52], as both RTs have similar enzymatic characteristics. Furthermore, it was shown that it was the nucleotide sequence surrounding the K65R codon, in particular a polyadenine stretch terminating at the position which is mutated in the lysine to arginine substitution, that is responsible for the difference in selection [53,54]. Because of the sequence of the K64 codon (AAA in clade C and AAG in clade B), this polyadenine stretch is present in subtype C, but not subtype B RT and increases the frequency of substitution by a mechanism of template-dependent translocation [55]; the RT pauses at the end of the polyadenine stretch, and the primer-template become misaligned. A nucleotide is added to the end of the misaligned primer, the primer and template strands realign, and the resulting point mutation leads to the K65R substitution (see Figure 2).

In a situation that mirrors that found in the subtype B virus, the presence of TAMs also reduces selection of K65R in the subtype C virus (although not to the extent seen in subtype B). Thus, the treatment regimen would be expected to influence the selection of K65R; initial treatment with AZT followed by tenofovir may result in a lower K65R frequency than the use of tenofovir as a front-line therapeutic [56]. Indeed, the clinical situation is clearly complicated by factors other than subtype differences. Some studies show extensive selection of K65R in subtype C virus following treatment with didanosine (up to 30% K65R) [57] following failure of first-line treatment with a combination NRTI/NNRTI combination therapy [58,59]. However in another study of HIV-1 subtype C pediatric patients from Botswana, all of whom had failed first-line therapy, K65R was observed at a low frequency (<5%) [60].

In addition to the increased frequency of the K65R substitution as a result of drug pressure, there is evidence that the mutation may be more prevalent in drug-naïve subtype C infected patients than in patients infected with subtype B [61]. Ultra-deep sequencing was used to determine the proportion of a patient’s viral load that contained the K65R substitution. Excluding subjects that likely had the mutation as a result of a transmitted resistant variant, 35.7% (10 of 28) of patients with subtype C HIV-1 infection had a low level of K65R variants, compared to only 2.2% (2 of 92) in subtype B [62]. However, as was the case for K65R prevalence following treatment, the situation in treatment-naïve patients is also complicated; in a second study that aggregated mutation rates from a series of studies of treatment-naïve patients, K65R was found to be very rare (0.1%) and, interestingly, differences in prevalence between subtypes was not significant [63].

## 8. Summary and Conclusions

The K65 is one of the few key residues in the catalytic site of HIV-1 RT that is mutated (to an R residue) in patients receiving NRTIs. The K65R mutation confers resistance to most NRTIs. It is observed rarely in subtype B infections and the highest occurrence of 5% is observed in the case of Tenofovir-treated individuals. It appears more frequently and rapidly in subtype C HIV-infected individuals on Tenofovir therapy. The K65R mutation reduces both the catalytic efficiency and the mutation rate of HIV-1 RT *in vitro* and decreases the effect of other NRTI resistance mutations, such as TAMs. X-ray crystallographic studies of K65R mutated HIV-1 RT in complex with template-primer and dNTP or tenofovir has provided critical insights to explain the biochemical properties of K65R RT.
